# Bacilli Carriers and Their Relation to Public Health

**Published:** 1911-06

**Authors:** K. R. Collins

**Affiliations:** Atlanta, Ga.


### BACILLI CARRIERS AND THEIR RELATION TO PUBLIC HEALTH.

K. R. Collins, M.D., Atlanta, Ga.

It is only within recent years that the full significance of bacilli carriers and their relation to the spread of infectious diseases has been recognized, but the importance of the place that this factor holds in the dissemination of disease is being stressed more and more.

Human beings, insects or animals may become carriers of the causes of certain diseases. The scope of this paper, however. will be limited to human carriers.

Carriers may be divided into three classes:

1st.—Permanent Carries: This class is confined to such persons who have never had the disease which is produced by the organism for which they act as host.

2nd.—Temporary Carriers: Such persons who, coming in close contact with a patient suffering with an infectious disease, acquire temporarily the organism relating to the disease.

3rd.—This class consists of convalescents from certain infectious diseases who continue after complete recovery to maintain for varying periods of time the organism causing the disease

The causes of all diseases of an infectious nature will not be transmitted by the three means mentioned above, some falling only under one, others two, and again, others three of the classes respectively.

We have more certain facts concerning the part played by human carriers, especially with reference to permanent carriers and contacts in the spread of typhoid fever and diphtheria, than any other of the contagious diseases.

First, as to carriers of the diphtheria bacilli, we find, according to Grahame Smith and others, that from ten to fifty per cent. of the contacts have been found to harbor the diphtheria bacilli in the throat and nasal passages for different periods of time. The percentage of persons acquiring the bacilli under these conditions will, of course, be affected by the precautions observed in the hygiene of the sick room. The greater care taken the less will be the danger to those in attendance on the patient and to persons with whom they come in contact, even after the persons examined harbored the virulent diphtheria bacillus.

Under the third class the convalescent individuals have been found to carry the active bacilli anywhere from a few days to six hundred and sixty-nine days after the disappearance of the membrane. All of these cases are more of a menace to the public than the patients during the height of the disease, because proper precautions are observed to prevent the spread of contagion at this time.

It might be well to state here something of the nature of diphtheria bacilli and allied organisms, for the reason that too often the morphology of the bacillus alone is relied upon in making a diagnosis, exceptionally such a diagnosis may be in error. There are a number of organisms that resemble the typical form of the diphtheria bacilli, and also its pleomorthic forms. In such cases where there is any doubt as to the diagnosis it is advisable to resort to the animal test. This takes some three or four days, and is not practical where antitoxin is indicated, but it gives valuable information as to the proper procedure for quarantining and disinfecting. Diphtheria bacilli may be divided into two classes, which are homologous in their morphology, but differ in their power of toxin production. They are designated as virulent and non-virulent. Williams and others, after years of observation, failed to convert a virulent into a non-virulent organism or vica versa. Park and Beebe found 80% virulent bacilli, and 15% non-virulent organisms in suspected cases. In normal persons 18% of virulent bacilli were found, while about .2% were non-virulent. The virulent organism is very readily distinguished from the non-virulent by its effect upon the guinea pig, hence the advisability of resorting to this test in all cases where diphtheria bacilli are found in normal throats or in individuals not presenting the typical symptoms of diphtheria.

Aside from the non-virulent form of the diphtheria bacillus, there are a few organisms which closely resemble the diphtheria bacillus in some of its unusual forms, but these differ from it in cultural characteristics and in the production of toxins. Such bacilli as the Hoffman bacilli and the bacillus Xerosis is frequently found in the conjunctiva, and occasionally, according to some authorities, has been the cause of slight pathological condition, others deny, however, its ability to produce the slightest degree of irritation. This is a fact to be considered in conditions of the conjunctiva which seem diphtheritic in appearance.

It is a well-known fact that convalescents from typhoid fever may carry the organisms for a few weeks to twelve or eighteen months after recovery. Such cases are a menace to the public, unless they can be made to understand the danger of communicating the disease to others, and can be thoroughly trained in taking the necessary precautions. It seems to me possible that such convalescence especially among school children of the age to attend school at a distance, where the dormitory system prevails, may be responsible for many outbreaks of typhoid fever in schools and colleges. A careful history from each child upon being entered at the beginning of every fall term, and examinations made when necessary, might be a useful method to pursue in preventing many epidemics of infectious diseases occurring throughout the year.

In regard to contacts in the case of typhoid fever we have no accurate knowledge corresponding to contact cases of diphtheria, but it would be perfectly possible for an individual to acquire temporarily the organisms, and become a means of transmission of the disease.

In regard to permanent carriers of typhoid fever:—Several cases have been reported where the individual without ever having the disease as far as could be ascertained, have acted as host to this organism, the dejecta presenting almost a pure culture at times. You may be familiar with the case of a cook reported by Dr. Soper of New York City. He found that typhoid fever developed in nine families shortly after this woman came into the house. In these nine families some twenty cases of typhoid fever developed with two deaths. The families did not all live in one city. Upon examination of the feces of this woman almost a pure culture of the typhoid facillus was found. She was then isolated and kept under observation for two years. Occasionally there would be a few days when very few of the organisms or none at all could be found upon examination, but the greater part of the time the bacillus typhosus surplanted almost all other organisms in the stools. This organism was virulent at all time. The woman gave no history of ever being ill, no evidence of infection of the gall bladder could be obtained; all treatment failed to eliminate the organisms. After being confined by the authorities for something over two years, she was released with instructions to report at intervals to the health department and to observe precautionary measures, and was not allowed to again follow her vocation as a cook.

The British Government reports such another case in India. The woman gave no history of ever having had an attack of typhoid fever, but acted as host to this organism, and some eight or ten cases of this disease developed at different times within her own household. The German authorities have tried in some cases to remove the cause by operation upon the gall bladder, assuming that this has become the seat of infection, but their success has not been such as to justify this procedure being universally adopted. There are a number of other diseases which are carried in the same way; that is, by the individual acting as host without being themselves infected. This has been demonstrated in a case of cerebrospinal meningitis.

In conclusion, it would seem advisable, especially in cases of epidemics of various infectious diseases, to consider the possibility of human carriers, and to give instructions to those recovering from infectious diseases how to protect others against the disease with which they have been infected and to examine contacts.

---

### SURGICAL SUGGESTIONS.

"Non-bacterial" pyuria is very suspicious of tuberculosis.

In nephrectomy for hypernophroma it is very important to remove the renal vein.

The transperitoneal route, with retraction of the colon, affords the best exposure for neoplasm.

In case of urinary extravasation from rupture of the urethra, make no attempt to pass a catheter, and waste no time in incising the edamatous area, but perform perineal urethrotomy at once.

In pyuria of prostatic origin—as shown by massage—if there has been no recent infection and, especially, if the pus is germ-free, prostatic calculii should be thought of. A skiagraph will determine their presence or absence.

If a hernia suddenly becomes irreducible, advise prompt operation; if the patient vomits, even once, insist upon it!

Don't jump to the conclusion that a benign stricture of the rectum is syphilitic. Gonorrheal proctitis causes dense cicatricial nifiltration.

Smearing vaseline over the buttocks in a rectal examination, scratching the furniture with basins, spattering the carpet with plaster of Paris, are some of the "little things" that will lead some patients to consult thereafter surgeons with more neatness, if less skill.

In any case in which catheterization is required, however careful the nurse or phsician, administer hexamethylenamine as a prophylactic against cystitis.

—*American Journal of Surgery.*

